# The Dark Tetrad: analysis of profiles and relationship with the Big Five personality factors

**DOI:** 10.1038/s41598-024-55074-w

**Published:** 2024-02-23

**Authors:** Raquel Gómez-Leal, Pablo Fernández-Berrocal, María José Gutiérrez-Cobo, Rosario Cabello, Alberto Megías-Robles

**Affiliations:** Faculty of Psychology, University of Málaga, Campus Teatinos, S/N, 29071 Málaga, Spain

**Keywords:** Human behaviour, Risk factors

## Abstract

The Dark Tetrad (DT) is composed of the traits of Narcissism, Machiavellianism, Psychopathy, and Sadism. Most studies analyzing the DT have employed a variable-centered approach, analyzing the traits separately. In the present study, we treat DT as a whole, adopting a person-centered approach. We analyzed different homogeneous subgroups of individuals characterized by specific DT profiles, aiming to examine their relationship with Big Five personality factors. A sample of 1149 participants (50.1% women, 18–79 years) completed The Short Dark Triad and the Assessment of Sadistic Personality instrument to assess DT, while the Mini-IPIP was used to assess the Big Five personality factors. Cluster analysis yielded five groups: Narcissism, Machiavellianism, Mean DT, Low DT, and High DT group. The main results showed that the High DT group was distinguished by higher levels of extraversion and lower levels of agreeableness and conscientiousness (compared with the Low DT group). Moreover, the Narcissism group was characterized by higher scores on emotional stability, openness to experience, and extraversion. Finally, distribution according to gender varied across DT groups (more men than women in the High DT group and the opposite in the Low DT group). Limitations and future lines of research are discussed.

## Introduction

The Dark Tetrad (DT) consists of four subclinical traits: Machiavellianism, Narcissism, Psychopathy, and Sadism^1,2^. The term DT comes from the widely studied *dark triad* concept^3^, to which the recent literature has added sadism due to its similarities with the other three traits^4,5^. These traits have a common core. For instance, individuals with high DT scores tend to be manipulative, show a significant lack of empathy, and present behavioral tendencies toward deception, aggressiveness, and self-promotion^2^. However, each trait also has its own characteristics. Machiavellianism is specifically associated with flattery, manipulation, and cynicism, and usually, these individuals engage in amoral behavior to impress others and are focused on their self-interest^6^. Narcissism is related to excessive grandiosity, superiority, admiration seeking, dominancy, and arrogance^7,8^. Psychopathy is a personality trait characterized by callousness, with non-empathetic and impulsive behavior, superficial charm, and criminal tendencies^9,10^. Finally, sadism is associated with the enjoyment of the suffering of others—physical and/or psychological^11^.

The terms DT and dark triad have attracted the attention of researchers in recent years^1,3^. As a result, numerous investigations highlight how individuals with high scores on these traits negatively impact both society and themselves through their typically high levels of aggressiveness, impulsivity, or the tendency to engage in norm-violating acts^2,12^. However, despite the ever-growing literature on DT, its relationship with a general personality framework such as the Five-Factor Model^13^, also known as the Big Five (BF) Model, remains unclear. The factors included in the BF Model (extraversion, agreeableness, conscientiousness, emotional stability, and openness to experience) have mostly shown mixed results or moderate correlations with the DT traits^2^.

Examination of the relationship between the constructs of DT and the BF model holds the potential to contribute to a more comprehensive understanding of human personality, impacting various fields of research and practice. From a clinical standpoint, identifying additional psychological issues associated with the DT can help to improve interventions for personality disorders and facilitate the development of more efficient treatments^14^. In the field of forensics and criminal investigation, understanding how the DT interacts with the BF factors is important for precise criminal profiling, particularly in cases of psychopathy linked to criminal and antisocial behavior^9^. In the workplace, knowledge of this relationship can inform personnel selection, team management, and the improvement of the work environment^15^. Given this backdrop, the present study aimed to provide an in-depth analysis of the relationship between DT and these personality factors.

Currently, the BF Model is the dominant theory in the personality research landscape^16^. This theory is widely recognized in personality psychology and has been extensively researched, with evidence supporting its relationship with multiple psychological constructs. The previous literature has also demonstrated the validity of this model^17^. Costa and McCrae^18^ originally described the following factors that constitute this theory: (1) Extraversion, associated with a preference to interact with others, to be energetic, and to show positive emotionality; (2) Agreeableness, defined by the tendency to be kind to others, trustworthy, altruistic, and compassionate; (3) Conscientiousness, associated with behaviors such as dependability, organization, punctuality, and purposefulness; (4) Emotional stability, associated with low levels of anxiety, worry, and behavioral inhibition; and (5) Openness to experience, associated with being imaginative, curious, and flexible. The components of this theory are considered universal and are, therefore, found in many cultures.

Although no research has jointly examined the relationship between the BF Model and DT, several studies have explored the link between the BF factors and some DT personality traits separately (e.g., with Machiavellianism and psychopathy^19^; with psychopathy^20^; with narcissism^21^. In this regard, the existing literature has yielded the following results for each DT trait:

- *Machiavellianism* The findings associating this DT trait with extraversion are contradictory. While some studies have found a positive relationship since Machiavellianism can have a social aspect, such as achieving goals through interpersonal maneuvering^22^, other studies reported no such relationship^2,23^. Machiavellianism has been negatively associated with agreeableness due to the associated manipulative characteristics and tendency to achieve goals over and above interpersonal relationships^24^. Although people with high Machiavellianism scores appear to be self-disciplined and achievement-oriented, studies examining the relationship between this trait and conscientiousness have produced mixed results^15,23^. And while the previous literature shows the extent to which certain depressive symptoms and higher levels of anxiety are linked to Machiavellianism^25,26^, investigations concerning emotional stability have yielded mixed results^27–29^. Finally, previous studies have found no evidence of an association with openness to experience since this DT trait is not related to characteristics such as creativity, imagination, or flexibility^6,30^.
- *Narcissism* The literature consistently shows that people with high levels of narcissistic traits display extraverted behaviors, such as talking in a group, socializing, and a tendency toward being energetic^31^. In addition, the literature has shown that they perceive themselves as extroverts^32^. Previous studies have also reported a negative relationship between this trait and agreeableness, which could be because individuals with high levels of narcissism are characterized by certain behaviors that would be expected to counteract this trait, such as not caring about the opinions and feelings of others^33,34^. Although individuals with high scores in this trait are self-disciplined and achievement-oriented, researchers have found mixed results when examining its relationship with conscientiousness^30,35^. The fact that the narcissistic trait is related to low levels of anxiety and depression symptoms makes it more likely that there is a positive association between this trait and emotional stability^26,36^. However, studies have found mixed results^21,37^ depending on the type of narcissism analyzed (e.g., grandiose vs. vulnerable). Finally, some authors have reported a positive relationship with openness to experience, since people with high scores in this trait have characteristics compatible with this factor, such as high creativity^38^.
- *Psychopathy* This trait has been negatively related to agreeableness and conscientiousness (see the meta-analysis by^39^). This relationship may be due to the fact that certain characteristics of psychopathic traits would counteract high scores on these two BF factors, such as lack of remorse, no respect and a disregard for others, or social manipulativeness^39,40^. Concerning extraversion, there are mixed results^15,41^. Thus, while people with high scores on this trait are characterized by a superficial charm that can lead them to be extroverted, such individuals experience low quality interpersonal relationships^39^. The results regarding emotional stability are also mixed^20,42^, which could be due to the fact that psychopathy traits are related to characteristics associated with high neuroticism, such as poor behavioral control, but also to characteristics associated with more emotional stability, such as low nervousness^43^. Finally, previous studies have reported contradictory results concerning the relationship with openness to experience^15,30^ since people with high psychopathic traits have characteristics compatible with this factor, such as active imagination, but also others that are incompatible, such as low openness to feelings^34,40^.
- *Sadism* The literature studying the relationship between sadism and the BF personality factors is scarce. Kowalski et al.^44^ conducted a small meta-analysis with nine studies, concluding that sadism is related to low agreeableness and low conscientiousness. These results could be due to the fact that people with high sadism scores show certain characteristics (such as low patience, indulgence, cooperative and moral behaviors and high impulsivity) that are incompatible with high levels of these factors^45^. Moreover, Kowalski et al.^44^ found a positive relationship with extraversion and a negative relationship with emotional stability although these relationships were statistically weak. The association with extraversion is unlikely to be strong since people with high traits of sadism—although disinhibited—experience a lack of positive interpersonal relationships^46^. Regarding emotional stability, previous studies have found a negative (or null) relationship between these two constructs, explaining these results by the fact that the absence of anxiety or depression are not main characteristics of sadism, although these individuals present a low level of emotion regulation^11,47,48^. Finally, the previous literature has reported mixed associations between sadism and openness to experience without a clear explanation for these findings^44,49^.

In summary, evidence suggests that the four traits of the DT can be related to low agreeableness. Regarding extraversion, while previous studies have found a positive relationship with narcissism and sadism, its relationships with the other DT traits are unclear. In addition, there appears to be a positive relationship between narcissism and open to experience, and a negative relationship between the DT traits of sadism and psychopathy and conscientiousness. For the rest of the relationships, previous studies have found mixed results and thus no firm conclusions can be drawn regarding associations between the constructs.

The literature reviewed offers a comprehensive overview of the relationship between DT and BF factors. However, all these studies have examined these traits in isolation, and none have analyzed the DT as a unified construct. The variable-centered approach employed in these studies concentrates on explaining individual relationships between variables and isolating psychologically meaningful behavioral characteristics in which individuals consistently differ. In contrast, our present study adopts a person-centered approach, delving into the examination of individual personality patterns or profiles composed of various traits^50,51^. This approach offers greater sensitivity to individual differences, recognizing that the relationship between DT and BF can vary significantly from one person to another depending on the unique combination of traits and factors. Using the person-centered approach, we identified different homogeneous subgroups of individuals characterized by specific profiles of Machiavellian, narcissistic, psychopathic, and sadistic scores allowing us to (1) categorize individuals into common subgroups, distinguishing, for example, those individuals characterized by high scores in the four DT traits from other profiles that only show high scores in some of the DT traits; and (2) examine the relationships between these subgroups and BF factors, which could help to resolve the discrepancies found in the previous literature and contribute to a better understanding of the personality characteristics associated with the DT.

It is also important to consider the fact that previous studies have revealed gender differences in most of the aforementioned variables. For example, the four DT traits are more predominant in men than women^31,52^. These differences are particularly evident in psychopathy and sadism, both in clinical and community samples^52,53^. With respect to gender differences in the BF personality factors, research has revealed that men score higher than women in emotional stability, while women score higher than men in agreeableness and extraversion^54^. These findings emphasize the importance of considering gender differences in the present investigation.

The main objective of this study was to clarify the relationship between DT and the BF personality factors, but unlike previous studies, we used a person-centered approach. To this end, we identified subgroups of individuals with different DT profiles using cluster analyses. We anticipated identifying a high DT group, a low DT group, and a group with intermediate scores. Additionally, we explored the possibility of identifying other different profiles where only one or some of the DT traits are highlighted. Based on the reviewed literature, we expected to find that (1) profiles with high scores on all the traits of DT are characterized by low agreeableness, (2) profiles with high scores in psychopathy and sadism traits, but average or low scores in the rest of the DT traits, are associated with less conscientiousness, (3) profiles with high scores in narcissism and average or low scores in the rest of traits are associated with more openness to experience and extraversion, and, (4) regarding gender differences, based on previous studies we expect to replicate the findings indicating that profiles with higher DT scores are more prevalent in men than women, while profiles with low DT scores will be more prevalent in women. Additionally, we hypothesize that men, compared with women, will score higher in emotional stability and lower in agreeableness and extraversion.

In addition to these hypotheses, there are other potential associations between DT and personality that lack clarity in the existing literature, such as the association between extraversion and both psychopathy and Machiavellianism. Adopting an exploratory approach, our study aims to investigate these relationships and address the inconsistencies in the current literature.

## Method

### Participants

The study participants were 1149 adults form a community sample (M_age_ = 36.30, SD = 14.57, ranging from 18 to 79 years; women = 50.1%). Participants were recruited through a snowball sampling technique with the support of students from the University of Málaga, Spain. This method was chosen due to its cost-effectiveness compared to other sampling approaches, enabling a wider reach within a relatively short period. Moreover, participants recruited through this method are often more willing to participate, possibly because they feel more at ease being referred by trusted individuals in their social circles^55^. All participants gave signed informed consent prior to completing the online survey and they were assured of the anonymity of the collected data. The ethical guidelines of the Helsinki declaration were followed. The study protocol was approved by the ethics committee of University of Málaga (approval number: CEUMA 14-2019-H) forming part of this research project (B1-2021_10).

### Procedure and instruments

Questionnaires were administered through the online platform LimeSurvey (http://limesurvey.org). Dark Triad traits were assessed using the Spanish version of The Short Dark Triad, the sadism trait was assessed by the Spanish version of Assessment of Sadistic Personality instrument, and the BF factors of personality were assessed using the Mini International Personality Item Pool scale. Participants needed between 20 and 25 min to complete these instruments.

*The Short Dark Triad* (*SD-3*;^56^) is a 27-item self-report questionnaire measuring the DT traits with 9 items each (Machiavellianism, narcissism, and psychopathy). Participants were asked to indicate their level of agreement with the statements on a five-point Likert scale, where 1 = *strongly disagree* and 5 = *strongly agree*. The SD-3 Spanish version^57^ reported reliability indices ranging between *α* = 0.61 and *α* = 0.80. In our study, the internal consistency ranged between *α* = 0.67 and *α* = 0.77.

*The Assessment of Sadistic Personality* (*ASP*;^45^) is a 9 item self-report questionnaire measuring sadism. We used the Spanish version of this scale^52^. The response format is the same as the *SD-3* scale (Likert scale from 1 = *strongly disagree*, to 5 = *strongly agree*). The reliability reported by^45^ was *α* = 0.83. In our study, the internal consistency was *α* = 0.86.

*The Mini International Personality Item Pool* (*Mini-IPIP*,^58^) is a self-report questionnaire used to assess extraversion, agreeableness, conscientiousness, emotional stability, and openness to experience. This instrument has 20 items answered on a five-point Likert scale (where 1 = *strongly disagree* and 5 = *completely agree*). We employed the Spanish version of the instrument^59^. This scale has shown to have acceptable psychometric properties where alpha values ranged from 0.69 to 0.81. In our study, the internal consistency ranged between *α* = 0.70 and *α* = 0.85.

### Data analysis

We first calculated descriptive statistics and explored the gender differences using *t*-tests for independent samples. Second, Pearson's correlations were conducted between DT traits and the BF personality factors. Third, a hierarchical cluster analysis using Ward’s method and the Squared Euclidean Distance was conducted to generate different DT profiles based on the scores obtained on the four DT traits. We relied on the dendrogram and the agglomeration schedule to evaluate the number of clusters. In addition, a discriminant analysis through Wilks' Lambda test was used to confirm the significant differences between the identified clusters. Differences between men and women in each cluster were examined using the binomial test. Finally, differences between the DT profiles as a function of the BF personality factors were tested using a multivariate analysis of covariance (MANCOVA). The BF personality factors were introduced as dependent variables, the DT profiles as independent variables, and gender and age as covariates. Tukey’s honestly significant difference (HSD) test was used for post-hoc comparisons when significant main effects were observed. All analyses were conducted using SPSS 24.0 (IBM Corporation, Armonk NY, USA) and Statistica 8 (StatSoft, Inc., USA).

## Results

Descriptive statistics and Student’s *t*-test for independent samples for all study variables are presented in Table 1. The kurtosis and skewness values were within the acceptable range of − 2 to 2, indicating that the variables were well-distributed and approximated to a normal distribution. Men scored significantly higher than women on all DT traits, emotional stability, and openness to experience, while agreeableness and conscientiousness scores were significantly higher for women than men (*p*s < 0.05; effect sizes ranging between small and medium).

**Table 1:** Means, standard deviations (SD), and *t*-tests for gender differences in all the study variables.

	Total sample		Men		Women		Gender differences	
	Mean	SD	Mean	SD	Mean	SD	t	Cohen’s d
Machiavellianism	24.10	6.02	25.55	5.86	22.65	5.81	8.43	0.49**
Narcissism	24.09	5.05	24.95	4.81	23.22	5.16	5.87	0.35**
Psychopathy	17.03	5.04	18.54	5.03	15.51	4.54	10.68	0.63**
Sadism	13.90	5.40	15.49	5.89	12.31	4.34	10.41	0.61**
Extraversion	2.76	0.94	2.75	0.93	2.78	0.95	− 0.49	0.03
Agreeableness	3.69	0.83	3.42	0.80	3.96	0.77	− 11.72	0.69**
Conscientiousness	3.49	0.86	3.37	0.82	3.61	0.88	− 4.73	0.28**
Emotional stability	2.79	0.82	2.99	0.77	2.59	0.81	8.71	0.51**
Openness to experience	3.25	0.93	3.31	0.90	3.18	0.97	2.38	0.14*

*p < .05, **p < .01.

Pearson's correlations between DT traits and BF personality factors (see Table 2) revealed significant positive correlations between all DT traits and extraversion (*p*s < 0.01; with a large effect size for narcissism and small effect sizes for the rest of the traits), between narcissism and emotional stability (*p* < 0.01; small effect sizes), and between the DT traits of narcissism and psychopathy and openness to experience (*p*s < 0.05; small effect sizes). Significant negative correlations were found between the DT traits of Machiavellianism, psychopathy and sadism and the factor of agreeableness (*p*s < 0.01: small effect sizes), and between the DT traits of psychopathy and sadism and conscientiousness (*p*s < 0.01; small effect sizes).

**Table 2:** Pearson's correlations among the study variables.

	2	3	4	5	6	7	8	9
1. Machiavellianism	0.31**	0.50**	0.42**	0.11**	− 0.20**	− 0.04	− 0.02	− 0.05
2. Narcissism	–	0.34**	0.29**	0.55**	0.05	0.05	0.15**	0.24**
3. Psychopathy		–	0.71**	0.18**	− 0.26**	− 0.25**	− 0.05	0.06*
4. Sadism			–	0.15**	− 0.28**	− 0.19**	0.01	0.04
5. Extraversion				–	0.32**	0.13**	0.17**	0.37**
6. Agreeableness					–	0.29**	0.03	0.35**
7. Conscientiousness						–	0.17**	0.11**
8. Emotional stability							–	0.19**
9. Openness to experience								–

*p < .05, **p < .01.

**Figure 1:**
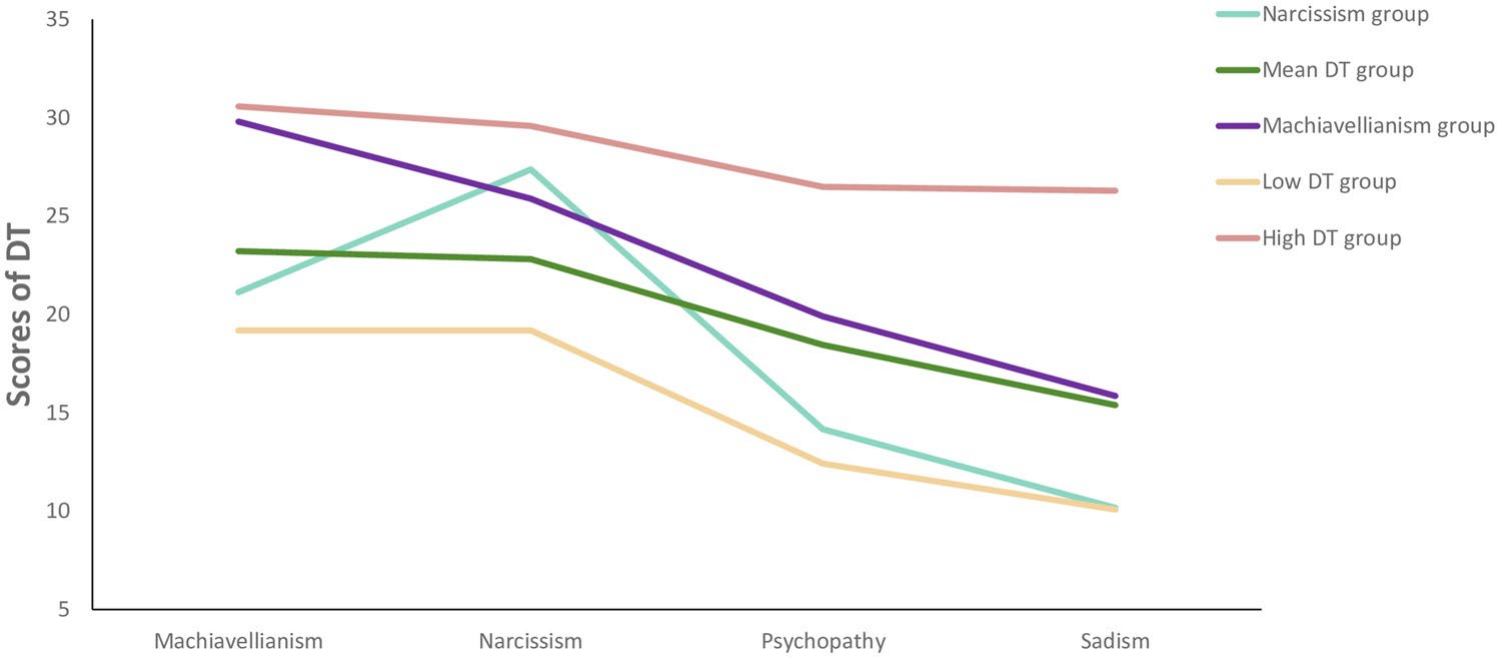
Cluster solution based on scores for DT traits.

The cluster analysis solution and DT scores for each cluster are represented in Fig. 1. Visual inspection of the dendrogram suggests five clusters, and the agglomeration schedule indicates that this five-cluster solution optimally fits the data. The first cluster was characterized by scores above the mean in the trait of narcissism, which we termed the Narcissism group (n = 224 [57.5% women; binomial test for gender distribution: *p* > 0.05]). A second cluster presented scores close to the mean in the four traits, which was termed the Mean DT group (n = 241 [47.7% women; binomial test: *p* > 0.05]). A third cluster was mainly distinguished by scores above the mean in the Machiavellianism trait and was thus labeled the Machiavellianism group (n = 317 [36.2% women; binomial test:* p* < 0.01]). A fourth cluster was characterized by scores below the mean on all traits, which was termed the Low DT group (n = 290 [69.6% women; binomial test:* p* < 0.01]). Finally, a fifth cluster with scores well above the mean in all traits was termed the High DT group (n = 78 [19.4% women; binomial test:* p* < 0.01]). A discriminant analysis (Wilks' Lambda test) revealed a good discrimination among clusters (*p* < 0.001), with 85% of cases correctly classified.

**Figure 2:**
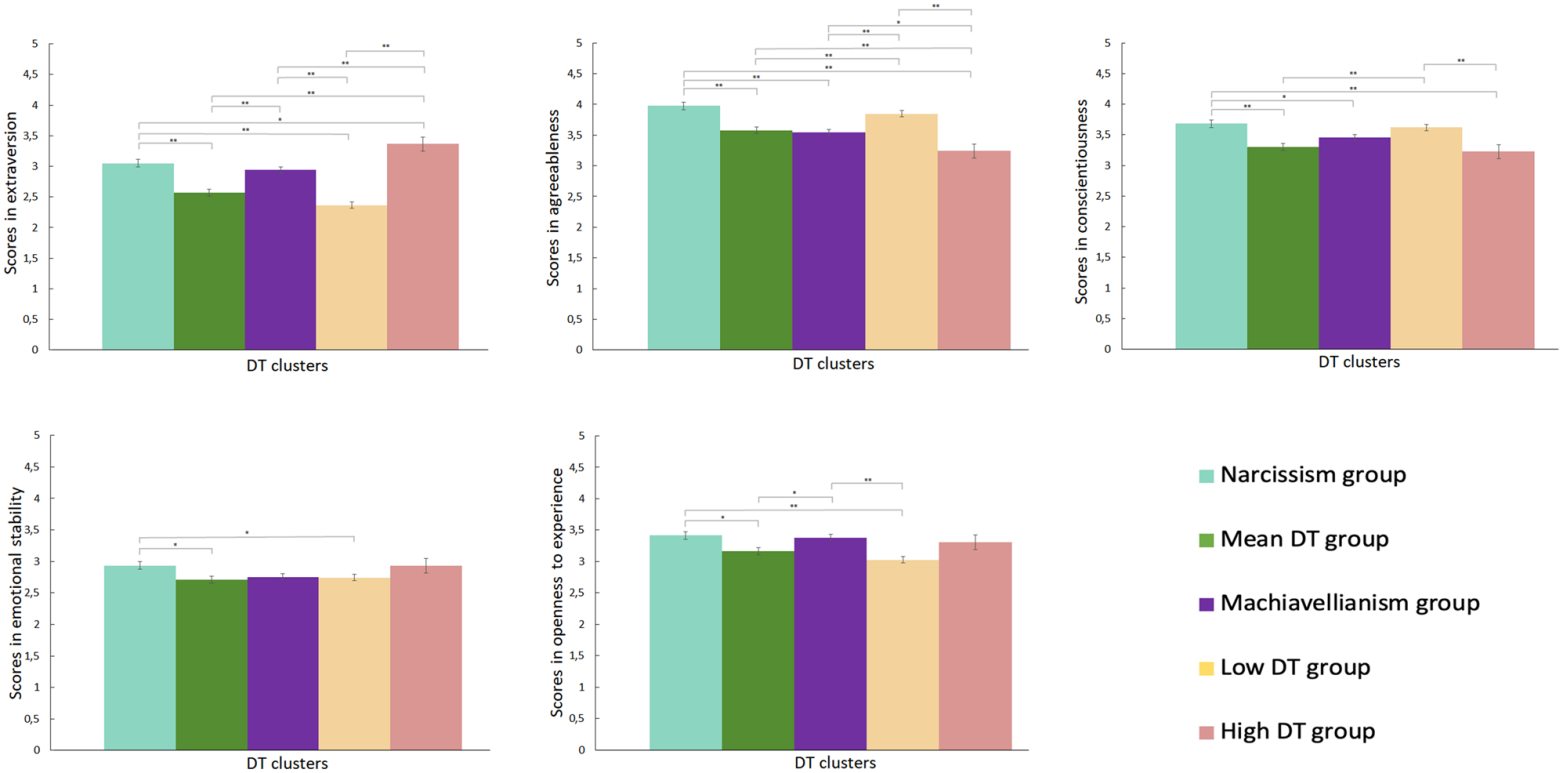
Group scores for personality traits. *p < .05, **p < .01.

Differences in the BF personality factors between the five identified DT clusters were tested using a MANCOVA (controlling for gender and age). Additionally, a MANCOVA was conducted where gender and age were introduced as independent variables, confirming that the DT cluster x gender and DT cluster x age interactions were not significant (*p* > 0.05). The MANCOVA revealed a significant multivariate effect of DT clusters (Wilk’s Lambda = 0.80, *F*[20, 3775] = 13.11, *p* < 0.001). Significant differences between clusters were observed for the five personality factors: extraversion (F[6, 1142] = 31.44, *p* < 0.001), agreeableness (*F*[6, 1142] = 37.15, *p* < 0.001), conscientiousness (*F*[6, 1142] = 12.19, *p* < 0.001), emotional stability (*F*[6, 1142] = 16.60, *p* < 0.001), and openness to experience (*F*[6, 1142] = 17.83, *p* < 0.001). Tukey’s HSD post-hoc comparisons for each personality factor revealed the following results (see Table 3 and Fig. 2): (a) with respect to extraversion, the Narcissism group showed higher scores than the Mean DT and Low DT groups (*p*s < 0.05). The Machiavellianism group showed higher scores than Low and Mean groups (*p*s < 0.05). The High DT group showed higher scores than the Narcissism, Mean DT, Machiavellianism, and Low DT groups; (b) with respect to agreeableness, the Narcissism group had higher levels than the Mean DT, Machiavellianism, and High DT groups, while the Machiavellianism group scored higher than the High DT group, the Mean DT group scored higher than the High DT group, and the Low DT group scored higher than the Mean DT, Machiavellianism, and high DT groups (*p*s < 0.05); (c) with respect to conscientiousness, the Narcissism group scored higher than the Mean DT, Machiavellianism, and High DT groups. Finally, the Low DT group had higher levels than the High DT and Mean DT groups (*p*s < 0.05); (d) with respect to emotional stability, only the Narcissism group scored higher than the Mean DT and Low DT groups (*p*s < 0.05); and (e) concerning openness to experience, we found that the Narcissism group and Machiavellianism group scored higher than the Mean DT and Low DT groups (*p*s < 0.05).

**Table 3:** Comparisons between DT groups for each of the Big Five factors.

	Narcissism group^a^ M (SD)	Mean DT group^b^ M (SD)	Machiavellianism group^c^ M (SD)	Low DT group^d^ M (SD)	High DT group^e^ M (SD)	*F*
Extraversion	3.05 (0.90)^bde^	2.57 (0.83)^ace^	2.94 (0.91)^bde^	2.37 (0.81)^ace^	3.37 (0.10)^abcd^	31.44*
Agreeableness	3.98 (0.80)^bce^	3.58 (0.80)^ade^	3.54 (.80)^ade^	3.85 (0.81)^bce^	3.24 (0.83)^abcd^	37.15*
Conscientiousness	3.68 (0.82)^bce^	3.30 (0.89)^ad^	3.45 (0.87)^a^	3.62 (0.82)^be^	3.22 (0.82)^ad^	12.19*
Emotional stability	2.93 (0.83)^bd^	2.71 (0.73)^a^	2.75 (0.84)	2.74 (0.86)^a^	2.93 (0.81)	16.60*
Openness to experience	3.41 (0.93)^bd^	3.16 (0.99)^ac^	3.38 (0.89)^da^	3.02 (0.98)^ac^	3.31 (0.95)	17.83*

Means with the lettered superscripts are significantly different from the DT groups corresponding to those letters (Tukey’s HSD post-hoc test). The letter representing each DT group (a, b, c, d, and e) is specified in the name of the group.

*p < 0.05.

## Discussion

The present study aimed to analyze the relationship between the DT and the BF personality factors through a person-centered approach, with the person —rather than a specific trait—being the unit of analysis. Cluster analysis identified five DT profiles based on levels of narcissism, Machiavellianism, psychopathy, and sadism. These profiles were (a) a Narcissism group characterized by scores above the mean for the narcissism trait; (b) a Machiavellianism group largely characterized by scores above the mean for the Machiavellianism trait; (c) a Mean DT group with mean scores across the four DT traits; (d) a Low DT group with scores below the mean on all DT traits; and e) a High DT group characterized by high scores across all DT traits.

In accord with our first hypothesis, the results indicate that the group with high scores on all DT traits is characterized by low agreeableness. As the previous literature has consistently demonstrated the negative relationship between agreeableness and DT traits^2,60–62^. People with low agreeableness share some of the basic aspects that characterize DT, such as antisocial characteristics, low levels of trust, tenderness, and altruism coupled with selfish, impatient, outspoken, and hard-hearted behaviors^63,64^. Low agreeableness in the high DT group seems to be associated with Machiavellianism, psychopathy, or sadism and not narcissism, according to the correlation analysis and the results shown by the Machiavellianism group. In fact, the Narcissistic group scored significantly higher in agreeableness than the rest of the groups. These results run counter to those reported in the literature, where previous studies have shown a negative relationship between narcissism and agreeableness^33,34^. Further exploration is needed to understand these discrepancies; however, it is possible that they could arise from the multifaceted nature of narcissism, which may include components linked to greater agreeableness. Narcissism is related to higher self-esteem^65^, potentially contributing to an improved ability to set boundaries and express opinions assertively^66^. Moreover, the manipulative behaviors associated with narcissism^67^ may include agreeable traits to attain objectives. In essence, agreeableness could serve individuals with narcissistic traits in reaching their goals.

Our second hypothesis predicted that profiles with high scores in psychopathy and sadism traits—but average or low scores in the rest of DT traits—would be associated with less conscientiousness. Although we found no specific group that was characterized exclusively by high scores in psychopathic and sadism traits since participants with high scores in sadism and psychopathy also showed elevated scores in other DT traits, the High DT group was the profile with the lowest levels of conscientiousness, significantly lower than the Low DT group and Narcissism group. These group differences, along with the fact that conscientiousness was significantly related only to the DT traits of psychopathy and sadism in the correlation analysis (although the effect size was small), suggest that the lower scores in conscientiousness observed in the High DT group could be associated with these traits, as predicted by our Hypothesis 2. This proposal is compatible with the previous literature^39,44^ and could be explained by the finding that individuals with higher scores in psychopathy and sadism are characterized by acting impulsively and irresponsibly, with an inability to wait for long-term rewards, and a failure to follow norms or customs^43,45^.

Our third hypothesis posited that profiles exhibiting elevated narcissism scores, coupled with average or low scores in other traits, would be associated with greater openness to experience and extraversion. In support of this hypothesis, we observed significantly higher levels of openness to experience in the Narcissism group compared with the Mean DT and Low DT groups. However, it should be noted that the correlation between narcissism and openness to experience revealed a small effect size. These results are consistent with the previous literature^2,68^ and could be related to the observation that individuals with a high narcissism score also tend to show high levels of self-reported curiosity and creativity^38,69^. Regarding extraversion, our results indicated higher levels of extraversion in the Narcissism group compared with the Mean DT and Low DT groups. Moreover, the correlation between narcissism and extraversion showed a large effect size. These findings could be attributed to the extraverted behaviors shown by individuals with high narcissism traits such as unrestraint, activity/adventurousness, socializing, or their own self-perception as extraverted people^30,31^, all of which are likely to help the individual feel valued, admired, and socially recognized.

Beyond the above hypotheses, our analyses revealed additional results that warrant discussion. First, the Narcissism group showed significantly higher levels of emotional stability compared with the Mean DT and Low DT groups. These findings are consistent with studies reporting a negative relationship between narcissistic traits and depressive and anxiety symptoms^26,36^. Second, significantly higher scores in extraversion were observed in the High DT group in comparison with the Narcissism, Mean DT, Machiavellianism, and Low DT groups. The higher scores in extraversion in the High DT group could be influenced not only by its association with narcissism but also by certain characteristics of the other DT traits. For instance, the relationship between Machiavellianism and certain social elements, such as the desire for good social relationships for later manipulation^22,58^ could contribute to higher extraversion scores. Additionally, higher scores on extraversion could be associated with psychopathy, given that individuals with high scores in this trait are often characterized by a tendency to show superficial charm^38^. Finally, sadism may be related to certain aspects of extraversion, such as disinhibited behavior^45^. These findings provide insights that could help clarify certain inconsistencies within the existing literature, which might be attributed to variations in sample characteristics in previous studies. For example, contrary to our findings, Jonason et al.^70^ reported a negative correlation between extraversion and psychopathy and found no correlation between extraversion and Machiavellianism. Another example is provided by^41^, who did not identify any association between extraversion and the traits of psychopathy and Machiavellianism. Notably, both studies used samples composed exclusively of university students.

Concerning gender differences, and in accord with our fourth hypothesis, the gender distribution varied depending on the DT group. In particular, the group characterized by high DT scores contained fewer women (19.4%) than men, while in the Low DT group there were more women (69.6%) than men. Moreover, and in line with previous studies^31,54^, men obtained higher scores on all the dimensions of DT and in emotional stability while women obtained higher scores in agreeableness. We also observed that men scored higher on openness to experience while women obtained higher scores on conscientiousness.

Although all these findings can help to advance our understanding of the relationship between the DT and the Big Five factors, several limitations should be considered. First, the questionnaires used in this research are self-reports and therefore may be influenced by social desirability^71^. In this type of sample, it is particularly important to employ more objective measures since individuals with high DT are characterized by behaviors such as lying, deceit, and manipulation^72^. Second, the cross-sectional methodology used in this study does not allow for establishing causality between the Big Five personality factors and the DT traits. Third, correlation analyses generally revealed small effect sizes, although these are consistent with previous literature^23^. Furthermore, the results of the MANCOVA corroborated our correlational findings, even controlling for relevant variables such as gender and age. Fourth, it would be interesting to replicate these results in specific samples, such as incarcerated individuals, or consider other aspects of the DT traits such as primary and secondary psychopathy^73^ or the grandiose and vulnerable facets of narcissism^74^. Finally, while the snowball method offers several advantages (see the method section), it is important to highlight some of its disadvantages when interpreting the results. These include potential selection bias, limited generalizability, and difficulty in controlling sample characteristics^75^.

## Conclusion

Identifying the different subgroups of individuals characterized by specific profiles of narcissism, Machiavellianism, psychopathy, and sadism traits can contribute to a better understanding of the DT concept and an analysis of how these traits combine with each other. We take advantage of this person-centered approach to examine the relationship between these DT profiles and the Big Five personality factors. The main findings of this study revealed that individuals characterized by high scores on the four DT traits show higher levels of extraversion and lower levels of agreeableness and conscientiousness (compared with individuals with low DT). In addition, individuals with high narcissism scores (regardless of the rest of DT traits) were characterized by higher scores on openness to experience, extraversion, and emotional stability.

An adequate conceptualization of personality traits is key to better assessment and diagnosis processes in mental health, criminal investigations, and workplace management. Such conceptualization must be guided by empirical research that identifies these traits, analyzes how they are related to each other, and studies how they behave with other variables^76^. We believe that the person-centered approach used in the present study can provide significant advantages in the study of DT and can inform the development of interventions aimed at preventing the negative consequences of DT for our society^2^.

## Data Availability

The datasets generated and/or analyzed during the current study are available from the corresponding author on reasonable request.

## References

[1] Bonfá-Araujo B, Lima-Costa AR, Hauck-Filho N, Jonason PK (2022). Considering sadism in the shadow of the Dark Triad traits: A meta-analytic review of the Dark Tetrad. Pers. Individ. Differ..

[2] Paulhus DL, Williams KM (2002). The Dark Triad of personality: Narcissism, Machiavellianism, and psychopathy. J. Res. Pers..

[3] Muris P, Merckelbach H, Otgaar H, Meijer E (2017). The malevolent side of human nature. Perspect. Psychol. Sci..

[4] Chabrol H, Van Leeuwen N, Rodgers R, Séjourné N (2009). Contributions of psychopathic, narcissistic, Machiavellian, and sadistic personality traits to juvenile delinquency. Pers. Individ. Differ..

[5] Paulhus DL (2014). Toward a taxonomy of dark personalities. Curr. Dir. Psychol. Sci..

[6] Collison KL, Vize CE, Miller JD, Lynam DR (2018). Development and preliminary validation of a five factor model measure of Machiavellianism. Psychol. Assess.

[7] Glover N, Miller JD, Lynam DR, Crego C, Widiger TA (2012). The five-factor narcissism inventory: A five-factor measure of narcissistic personality traits. J. Pers. Assess..

[8] Nenadić I, Lorenz C, Gaser C (2021). Narcissistic personality traits and prefrontal brain structure. Sci. Rep..

[9] Patrick, C. J. Psychopathy as masked pathology. in *Handbook of psychopathy* 3–21 (Guilford Press., New York, NY, 2018).

[10] Ju U, Williamson J, Wallraven C (2022). Predicting driving speed from psychological metrics in a virtual reality car driving simulation. Sci. Rep..

[11] Buckels EE, Jones DN, Paulhus DL (2013). Behavioral confirmation of everyday sadism. Psychol. Sci..

[12] Chester DS, DeWall CN, Enjaian B (2019). Sadism and aggressive behavior: Inflicting pain to feel pleasure. Pers. Soc. Psychol. Bull..

[13] McCrae RR, Buss DM, Cantor N (1989). Why I advocate the five-factor model: Joint analyses of the NEO-PI with other instruments. Personality Psychology: Recent Trends and Emerging Directions.

[14] Gómez-Leal R (2019). Relationship between the Dark Triad and depressive symptoms. PeerJ.

[15] Liang C, Huang Y (2015). A comparative study between the dark triad of personality and the big five. Canad. Soc. Sci..

[16] McCrae RR, John OP (1992). An Introduction to the five-factor model and its applications. J. Pers..

[17] Barrick MR, Mount MK (1991). The Big Five personality dimensions and job performance: A meta-analysis. Pers. Psychol..

[18] Costa PT, McCrae RR (1988). Personality in adulthood: A six-year longitudinal study of self-reports and spouse ratings on the NEO Personality Inventory. J. Pers. Soc. Psychol..

[19] Ashton MC, Lee K, Son C (2000). Honesty as the sixth factor of personality: Correlations with machiavellianism, primary psychopathy, and social adroitness. Eur. J. Pers..

[20] Borroni S, Somma A, Andershed H, Maffei C, Fossati A (2014). Psychopathy dimensions, Big Five traits, and dispositional aggression in adolescence: Issues of gender consistency. Pers. Individ. Differ..

[21] Furnham A, Hughes DJ, Marshall E (2013). Creativity, OCD, Narcissism and the Big Five. Think Skills Creat..

[22] Allsopp J, Eysenck HJ, Eysenck SBG (1991). Machiavellianism as a component in psychoticism and extraversion. Pers. Individ. Differ..

[23] O’Boyle EH, Forsyth DR, Banks GC, Story PA, White CD (2015). A meta-analytic test of redundancy and relative importance of the Dark Triad and five-factor model of personality. J. Pers..

[24] Jones, D. N. & Paulhus, D. L. Machiavellianism. in *Handbook of individual differences in social behavior* 93–108 (The Guilford Press., 2009).

[25] Al Aïn S, Carré A, Fantini-Hauwel C, Baudouin J-Y, Besche-Richard C (2013). What is the emotional core of the multidimensional Machiavellian personality trait?. Front. Psychol..

[26] Gómez-Leal R (2019). Relationship between the Dark Triad and depressive symptoms. PeerJ.

[27] Austin EJ, Farrelly D, Black C, Moore H (2007). Emotional intelligence, Machiavellianism and emotional manipulation: Does EI have a dark side?. Pers. Individ. Differ..

[28] de Vries RE, van Kampen D (2010). The HEXACO and 5DPT models of personality: A comparison and their relationships with psychopathy, egoism, pretentiousness, immorality, and Machiavellianism. J. Pers. Disord..

[29] Szijjarto L, Bereczkei T (2015). The Machiavellians’ “cool syndrome”: They experience intensive feelings but have difficulties in expressing their emotions. Curr. Psychol..

[30] Jakobwitz S, Egan V (2006). The dark triad and normal personality traits. Pers. Individ. Differ..

[31] Grijalva E, Harms PD, Newman DA, Gaddis BH, Fraley RC (2015). Narcissism and leadership: A meta-analytic review of linear and nonlinear relationships. Pers. Psychol..

[32] Holtzman NS, Vazire S, Mehl MR (2010). Sounds like a narcissist: Behavioral manifestations of narcissism in everyday life. J. Res. Pers..

[33] Campbell WK, Miller JD, Widiger TA, Costa PT (2013). Narcissistic Personality Disorder (NPD) and the Five-Factor Model: Delineating NPD, grandiose narcissism, and vulnerable narcissism. Personality Disorders and the Five-Factor Model of Personality.

[34] Samuel DB, Widiger TA (2008). Convergence of Narcissism measures from the perspective of general personality functioning. Assessment.

[35] Coleman G, Furnham A, Treglown L (2022). Exploring the Dark side of conscientiousness. The relationship between conscientiousness and its potential derailers: perfectionism and narcissism. Curr. Psychol..

[36] Spano L (2001). The relationship between exercise and anxiety, obsessive-compulsiveness, and narcissism. Pers. Individ. Differ..

[37] Zajenkowski M, Szymaniak K (2021). Narcissism between facets and domains. The relationships between two types of narcissism and aspects of the Big Five. Curr. Psychol..

[38] Goncalo JA, Flynn FJ, Kim SH (2010). Are two narcissists better than one? The link between narcissism, perceived creativity, and creative performance. Pers. Soc. Psychol. Bull..

[39] Lynam DR, Derefinko KJ, Patrick CJ (2006). Psychopathy and personality. Handbook of Psychopathy.

[40] Decuyper M, De Pauw S, De Fruyt F, De Bolle M, De Clercq BJ (2009). A meta-analysis of psychopathy-, antisocial PD- and FFM associations. Eur. J. Pers..

[41] Lee K, Ashton MC (2005). Psychopathy, machiavellianism, and narcissism in the five-factor model and the HEXACO model of personality structure. Pers. Individ. Differ..

[42] Daffern M, Gilbert F, Lee S, Chu CM (2016). The relationship between early maladaptive schema, psychopathic traits, and neuroticism in an offender sample. Clin. Psychol..

[43] Miller JD, Lynam DR (2015). Psychopathy and personality: Advances and debates. J. Pers..

[44] Kowalski CM, Di Pierro R, Plouffe RA, Rogoza R, Saklofske DH (2020). Enthusiastic acts of evil: The assessment of sadistic personality in polish and italian populations. J. Pers. Assess..

[45] Plouffe RA, Smith MM, Saklofske DH (2019). A psychometric investigation of the assessment of Sadistic Personality. Pers. Individ. Differ..

[46] Međedović J, Petrović B (2015). The Dark Tetrad. J. Individ. Differ..

[47] Greenfield DN (2023). Emotional intelligence in incarcerated sexual offenders with sexual sadism. J. Sex. Aggress..

[48] Greitemeyer T (2015). Everyday sadism predicts violent video game preferences. Pers. Individ. Differ..

[49] van Geel M, Goemans A, Toprak F, Vedder P (2017). Which personality traits are related to traditional bullying and cyberbullying? A study with the Big Five, Dark Triad and sadism. Pers. Individ. Differ..

[50] Howard MC, Hoffman ME (2018). Variable-centered, person-centered, and person-specific approaches. Organ Res. Methods.

[51] Morin AJS, Bujacz A, Gagné M (2018). Person-centered methodologies in the organizational sciences. Organ Res. Methods.

[52] Pineda D, Piqueras JA, Galán M, Martínez-Martínez A (2023). Everyday sadism: Psychometric properties of three Spanish versions for assessing the construct. Curr. Psychol..

[53] Cale EM, Lilienfeld SO (2002). Sex differences in psychopathy and antisocial personality disorder. Clin. Psychol. Rev..

[54] Weisberg YJ, DeYoung CG, Hirsh JB (2011). Gender differences in personality across the ten aspects of the Big Five. Front. Psychol..

[55] Sadler GR, Lee H-C, Lim RS-H, Fullerton J (2010). Research Article: Recruitment of hard-to-reach population subgroups via adaptations of the snowball sampling strategy. Nurs. Health Sci..

[56] Jones DN, Paulhus DL (2014). Introducing the short Dark Triad (SD3). Assessment.

[57] Pineda D, Sandín B, Muris P (2020). Psychometrics properties of the Spanish version of two dark triad scales: The dirty dozen and the short dark triad. Curr. Psychol..

[58] Donnellan MB, Oswald FL, Baird BM, Lucas RE (2006). The mini-IPIP scales: Tiny-yet-effective measures of the Big Five factors of personality. Psychol Assess..

[59] Martínez-Molina A, Arias VB (2018). Balanced and positively worded personality short-forms: Mini-IPIP validity and cross-cultural invariance. PeerJ.

[60] Jonason PK, Li NP, Webster GD, Schmitt DP (2009). The dark triad: Facilitating a short-term mating strategy in men. Eur. J. Pers..

[61] Miller JD (2010). Searching for a vulnerable dark triad: comparing factor 2 psychopathy, Vulnerable narcissism, and borderline personality disorder. J. Pers..

[62] Williams KM, Nathanson C, Paulhus DL (2010). Identifying and profiling scholastic cheaters: Their personality, cognitive ability, and motivation. J. Exp. Psychol. Appl..

[63] Costa PT, McCrae RR (1992). The Five-Factor model of personality and its relevance to personality disorders. J. Pers. Disord..

[64] Garcia D, Adrianson L, Archer T, Rosenberg P (2015). The dark side of the affective profiles. Sage Open.

[65] Bosson JK (2008). Untangling the links between narcissism and self-esteem: A theoretical and empirical review. Soc. Personal Psychol. Compass.

[66] Batmaz M, Buzlu S, Kutlu Y, Pektekin Ç (1996). Investigation of the levels of assertiveness and self esteem of last year nursing students at Istanbul University Health Services Occupational School’s Nursing Program. Nurs. Bull..

[67] Paulhus DL, Williams KM (2002). The dark triad of personality: Narcissism, Machiavellianism, and psychopathy. J. Res. Pers..

[68] Vernon PA, Villani VC, Vickers LC, Harris JA (2008). A behavioral genetic investigation of the Dark Triad and the Big 5. Pers. Individ. Differ..

[69] Kashdan TB (2013). Curiosity protects against interpersonal aggression: Cross-sectional, daily process, and behavioral evidence. J. Pers..

[70] Jonason PK, Kaufman SB, Webster GD, Geher G (2013). What lies beneath the dark triad dirty dozen: Varied relations with the big five. Indiv. Differ. Res..

[71] Anastasi A (1982). Psychological Festing.

[72] Lyons M (2019). The Dark Triad of Personality: Narcissism, Machiavellianism, and Psychopathy in Everyday Life.

[73] Levenson MR, Kiehl KA, Fitzpatrick CM (1995). Assessing psychopathic attributes in a noninstitutionalized population. J. Pers. Soc. Psychol..

[74] Ackerman RA, Donnellan MB, Robins RW (2012). An item response theory analysis of the narcissistic personality inventory. J. Pers. Assess..

[75] Magnani R, Sabin K, Saidel T, Heckathorn D (2005). Review of sampling hard-to-reach and hidden populations for HIV surveillance. AIDS.

[76] Stead R, Cynthia Fekken G, Kay A, McDermott K (2012). Conceptualizing the Dark Triad of personality: Links to social symptomatology. Pers. Individ. Differ..

